# Feasibility of using intermittent active monitoring of vital signs by smartphone users to predict SARS-CoV-2 PCR positivity

**DOI:** 10.1038/s41598-023-37301-y

**Published:** 2023-06-29

**Authors:** Nikola Dolezalova, Effrossyni Gkrania-Klotsas, Davide Morelli, Alex Moore, Adam C. Cunningham, Adam Booth, David Plans, Angus B. Reed, Mert Aral, Kirsten L. Rennie, Nicholas J. Wareham

**Affiliations:** 1 Huma Therapeutics Ltd., Millbank Tower, 21-24 Millbank, London, UK; 2 MRC Epidemiology Unit, Institute of Metabolic Science, University of Cambridge, Cambridge, UK; 3 Department of Infectious Diseases, Addenbrooke’s Hospital, Box 25, Cambridge, UK; 4 Department of Engineering Science, Institute of Biomedical Engineering, University of Oxford, Oxford, UK; 5 Department of Experimental Psychology, University of Oxford, Oxford, UK; 6 INDEX Group, Department of Science, Innovation, Technology, and Entrepreneurship, University of Exeter, Exeter, UK

**Keywords:** Epidemiology, Diagnostic markers

## Abstract

Early detection of highly infectious respiratory diseases, such as COVID-19, can help curb their transmission. Consequently, there is demand for easy-to-use population-based screening tools, such as mobile health applications. Here, we describe a proof-of-concept development of a machine learning classifier for the prediction of a symptomatic respiratory disease, such as COVID-19, using smartphone-collected vital sign measurements. The Fenland App study followed 2199 UK participants that provided measurements of blood oxygen saturation, body temperature, and resting heart rate. Total of 77 positive and 6339 negative SARS-CoV-2 PCR tests were recorded. An optimal classifier to identify these positive cases was selected using an automated hyperparameter optimisation. The optimised model achieved an ROC AUC of 0.695 ± 0.045. The data collection window for determining each participant’s vital sign baseline was increased from 4 to 8 or 12 weeks with no significant difference in model performance (F(2) = 0.80, p = 0.472). We demonstrate that 4 weeks of intermittently collected vital sign measurements could be used to predict SARS-CoV-2 PCR positivity, with applicability to other diseases causing similar vital sign changes. This is the first example of an accessible, smartphone-based remote monitoring tool deployable in a public health setting to screen for potential infections.

## Introduction

The use of telemedicine and mobile health technologies increased rapidly over the course of the COVID-19 pandemic. Smartphone applications were used for remote patient monitoring and delivering routine medical appointments^1–3^, to allow scientific research to take place remotely^4^, and to support ongoing clinical trials^5,6^.

Early detection of infectious diseases, such as COVID-19, would allow for timely isolation and thereby potentially reduce disease transmission. Throughout the study period, from August 2020 to April 2021, the COVID-19 case rates in the United Kingdom were relatively low, with a wave of infections peaking on 29th December 2020^7^. By 4th January 2021, the initial severe acute respiratory syndrome coronavirus 2 (SARS-CoV-2) variant was outnumbered by Alpha (B.1.1.7), which remained predominant until the end of this study^8^. These SARS-CoV-2 non-Delta variants were characterised by an incubation period with a median length of 5 days^9,10^ and a peak of infectivity 1 to 4 days prior to the onset of symptoms^11–13^. While the principal indicative symptoms were fever and respiratory-related, now it is recognised that the condition can manifest with a wide range of extrapulmonary symptoms^14^. Clinical presentation can range from asymptomatic to severe disease and death^15^. As SARS-CoV-2 can be detected in the upper respiratory tract about 2 days before the onset of symptoms^16^, vital sign data could be used for such early detection of physiological changes after infection but before symptom onset. If equally effective and accessible, this could replace the need for asymptomatic testing, while conferring the same benefits^17–19^.

Infection-triggered early inflammatory responses have been shown to provoke changes in resting heart rate^17,18,20–22^, oxygen saturation^23^, and body temperature^19,21^. These data could be entered into a smartphone app and employed at scale by early detection models for wide-ranging public health initiatives. A number of risk prediction or prognostic prediction models leveraging this data have been developed for SARS-CoV-2^17,18,24,25^. However, very few diagnostic models have been published^26^. These models have all been based on continuous passive wearable measurements, often using a single wearable device model. The limitations of this approach from a public health perspective include its scalability, owing to the financial cost of wearable devices, and the unaddressed necessity to harmonise outputs from different device models. In this pilot study, we used vital signs data intermittently collected via a smartphone app, without the use of a wearable, to investigate the feasibility of predicting respiratory disease, using the example of SARS-CoV-2 PCR positivity in a fully remote population-based cohort.

## Methods

### Participant recruitment and data collection

Participants were recruited from an ongoing observational cohort, the Fenland study. The Fenland study is a population-based cohort study of 12,435 participants born between 1950 and 1975. Participants were recruited from General Practice sampling frames in Cambridgeshire. Further details are published elsewhere^27,28^. The main aim of the Fenland COVID-19 study was to determine the prevalence of previous infection with COVID-19 in this known population-based cohort using three-monthly blood sample measures of SARS-CoV-2 IgG antibodies^28^. The Fenland COVID-19 app nested study aimed to investigate the progression of SARS-CoV-2 from the pre-symptomatic to the symptomatic stage using smartphone-acquired digital measures collected by a bespoke app, developed by Huma Therapeutics. It followed 2199 UK participants from 6th August 2020 for a minimum of 6 months and a maximum of 9 months. Ethical approval was provided by the South West-Cornwall & Plymouth Research Ethics Committee (REC reference 20/SW/0100). The Fenland Participant and Public Involvement (PPI) panel was involved in the planning, conducting, and reporting of the Fenland COVID-19 study. All participants provided written informed consent prior to taking part in the nested study, in accordance with the Declaration of Helsinki^29^.

### Collected data

SARS-CoV-2 PCR test results from the Second Generation Surveillance System (SGSS), the national reporting system across England, were obtained for all Fenland COVID-19 study participants during the study period. These contained all routine laboratory tests for SARS-CoV-2 infections from hospitals (patients and NHS staff) and community testing in the general population, both before and during the study period. In this analysis, we used the date of the first confirmed positive SARS-CoV-2 PCR test result to classify participants as having had a SARS-CoV-2 virus infection either before or during the study.

Dried blood spot samples were collected remotely by participants every 3 months during the study to determine the presence of SARS-CoV-2 antibodies. These were analysed for SARS-CoV-2 IgG antibodies using a commercial enzyme-linked immunosorbent assay (ELISA) targeting Spike (S2) and Nucleoprotein (N) from SARS-CoV-2 (Omega Diagnostics, UK), interfacing with the semi-quantitative Omega/Mologic SARS-CoV-2 IgG assay^28^. Results were classified as positive or negative, borderline results were considered negative.

All participants were asked to complete a baseline questionnaire at the onset of the study, containing information about any previous SARS-CoV-2 infections. Subsequently, on a monthly basis, participants completed questionnaires about changes in their health status and whether they had received a SARS-CoV-2 vaccination in the prior month, including the date and type of vaccination.

Three times per week, participants were asked to provide measurements of oxygen saturation levels, body temperature, and resting heart rate using a provided pulse oximeter (ChoiceMMed MD300C29), digital thermometer (Genial Digital Thermometer T12L), and their smartphone camera, respectively. Participants were asked to manually enter the results from the pulse oximeter and thermometer into the app. Resting heart rate was captured by the participant placing their finger over the camera on their smartphone for approximately 60 s^30^. For practicality, and to control diurnal variation, participants were asked to take all measurements in the morning after awakening. However, in analyses, we used all measurements irrespective of the time of day or, if multiple entries per day, the mean of the measurements.

At each measurement time point, participants were also asked to record whether they were experiencing any symptoms from a predetermined list, select “no symptoms”, and/or add other symptoms not in the list. The list of symptoms was updated regularly during the study as further symptoms were reported. For this analysis, the presence of any of the three core SARS-CoV-2 symptoms (fever, cough, and loss of taste and/or smell) identified at the time of the study were categorised as “yes” or “no”. In addition, two numerical features were extracted: number of the confirmed SARS-CoV-2 symptoms and number of any symptoms (from a longer list detailed in Supplementary Table 1) in the 7 days preceding the COVID-19 PCR test.

### Definition of model outcome

The machine learning task was designed as a binary classification of positive and negative SARS-CoV-2 PCR tests, using longitudinal records of vital signs as inputs. The positive class consisted of participants who had a positive SARS-CoV-2 PCR test during the study period. We chose the PCR positivity as evidence of SARS-CoV-2 infection, and not the antigen test, because PCR test is considered the gold standard in testing. The negative class consisted of a random sample of participants who during the study period both never had a positive antibody test, and had a negative PCR test that took place at least 28 days prior to the last antibody test. To remove seasonality effects on vital signs and ensure a balanced dataset, four negative cases were sampled for each positive case by matching the test dates (± 3 days).

### Participant inclusion/exclusion and data censoring

Participants of the Fenland App Study who completed the baseline questionnaire were included in this study. Exclusion criteria were a previous self-reported SARS-CoV-2 infection (“antigen test”) or a positive SARS-CoV-2 antibody test at the start of the study. We chose to exclude these participants as research at the time suggested that the immune response to a second infection may differ from the first infection. Additionally, there is a possibility that a negative PCR can follow a positive antigen test. For these reasons, we decided to exclude participants with reported positive self-sampling antigen tests and limit our study to the population with no positive antigen tests. Furthermore, participants with insufficient longitudinal vital sign data were excluded. Sufficient longitudinal vital sign data were defined as a minimum of one record of heart rate, oxygen saturation, and temperature during the week before a SARS-CoV-2 PCR test and at least two records in the 3 weeks prior to that time point.

Data collection on the smartphone app started on 6th August 2020 and the study closed on 30th April 2021. Participant-specific censoring was applied from (1) the time of withdrawal until the end of the study, (2) from 90 days before a positive SARS-CoV-2 antibody test until the end of the study, (3) from the day after a positive SARS-CoV-2 PCR test until the end of the study, and (4) from the day of SARS-CoV-2 self-reported vaccination for a duration of 5 days.

### Data pre-processing

Raw longitudinal vital sign data were cleaned as follows: non-physiological values were removed (< 89% and > 100% for oxygen saturation, < 40 and > 180 BPM for resting heart rate, and temperature measurements of < 35 °C). Resting heart rate values further than five standard deviations from the population mean were also removed.

The longitudinal data were then up-sampled to a daily frequency and linear interpolation was applied to fill in missing values between individual time points and values were also forward-filled after the last provided time point. Time points of interest were then filtered relative to the date of the SARS-CoV-2 test. All features were normalised by subtracting the participant’s mean and dividing by the participant’s standard deviation that was calculated on the training dataset prior to model training.

### Feature transformations

In addition to using the raw vital sign data, we performed several transformations on the data. These involved splitting the pre-processed longitudinal data into baseline data (e.g. − 28 days to − 7 days prior to test) used to calculate the baseline/normal representation for each individual, and transformed data (e.g. − 6 days to 0 days prior to test) used as transformed inputs for classification.

The first transformation was a z-score: for each day of the transformed data period, a z-score was calculated using the mean and standard deviation of baseline data (separate for each vital sign). For the next two transformations, we used anomaly detection algorithms.

The Isolation Forest algorithm, implemented via the python scikit-learn library^31^, was fitted on the baseline data for each participant individually. For each day of the transformed data period, an anomaly score was predicted and used as the multivariate feature.

Next, a Vector AutoRegression (VAR) model, implemented via the python statsmodels library^32^, was fitted on the baseline data for each participant individually and data was forecasted for each day of the transformed period. This forecasted data was then compared to the actual data and reconstruction errors were used as the transformed features (one for each vital sign and a summed multivariate feature).

Finally, we created an additional feature based on each of the above which took a maximum value of each transformed feature over the transformed time period (e.g. 7 days). The list of all transformed features used in the model can be found in Supplementary Table 1.

### Model selection and evaluation pipeline

Each experiment was configured based on the input features (e.g. heart rate, oxygen saturation, and temperature), the decision on whether to use raw or transformed features, the number of weeks of data provided to the model (e.g. 4, 8, or 12 weeks) and the end of the longitudinal data stream (e.g. –3, –2, –1, 0 days before the SARS-CoV-2 test). All further parameters were optimised by the pipeline described below.

If raw features were used in the model, the feature space was reduced by recursive feature elimination (using the RFECV class of scikit-learn library, to create a support vector classifier with linear kernel and balanced class weights, evaluated over three stratified folds by the area under ROC curve, with a minimum of five features to be selected). For transformed features, features were selected in sets based on transformation operation by Optuna^33^ hyperparameter optimization procedure described below.

The optimisation pipeline was further comparing three classification algorithms: logistic regression, random forest, and support vector classification; all implemented in the scikit-learn library. Balanced class weights were used for all three models and the maximum number of iterations was increased to 4000 for logistic regression. Other parameters of the models were either optimised during the hyperparameter search or kept as default.

Optimisation was performed using Tree-Structured Parzen Estimator (TPE) from the Optuna library and the details of the search space are provided in Supplementary Table 2. Based on the size of the search space, 100 and 500 Optuna trials were performed for raw or transformed features, respectively. Optimisation was based on maximisation of the area under the ROC curve score (AUC ROC score). Random seeds were set to allow for reproducibility of the results.

To obtain an unbiased estimate of the model performance, a nested cross-validation procedure was used. The dataset was split into five outer folds (stratified on outcome), for each of which the best model was selected after a separate feature selection and hyperparameter optimization were evaluated on the five inner folds. The performance of this best model was then evaluated on the unseen holdout set for the respective outer fold^34^.

To explore if it was possible to predict PCR test positivity in the period before the test date, models were trained using the previously determined optimal model hyperparameters and data from different days relative to the positive test.

Finally, we also investigated if it was possible to detect vaccination events, under the assumption that a vaccination would cause a change in vital signs. Models were constructed as described previously, but used the date of first COVID-19 vaccination instead of the positive test date for the positive class. Participants used as negative cases were not vaccinated during the window. Positive and negative cases were also matched on age and sex.

Besides the AUC ROC score, accuracy, precision, and recall were recorded for each result of the cross-validation pipeline. Further, we obtained results through the generation of confusion matrices, ROC AUC curves, and precision-recall curves (PR curves). These were all calculated according to the implementation in the scikit-learn library.

### Statistical analysis and visualisations

Demographic comparison of positive and negative classes was performed with the help of the python tableone library^35^. Statistical comparisons were obtained by performing a two-sample t-test for continuous variables and a chi-squared test for categorical variables, evaluated on a significance level of 0.05.

All visualisations were generated using the matplotlib^36^ and seaborn^37^ libraries. A full list of all python libraries and their versions used in this study can be found in Supplementary Table 3.

## Results

### Study population

**Figure 1:**
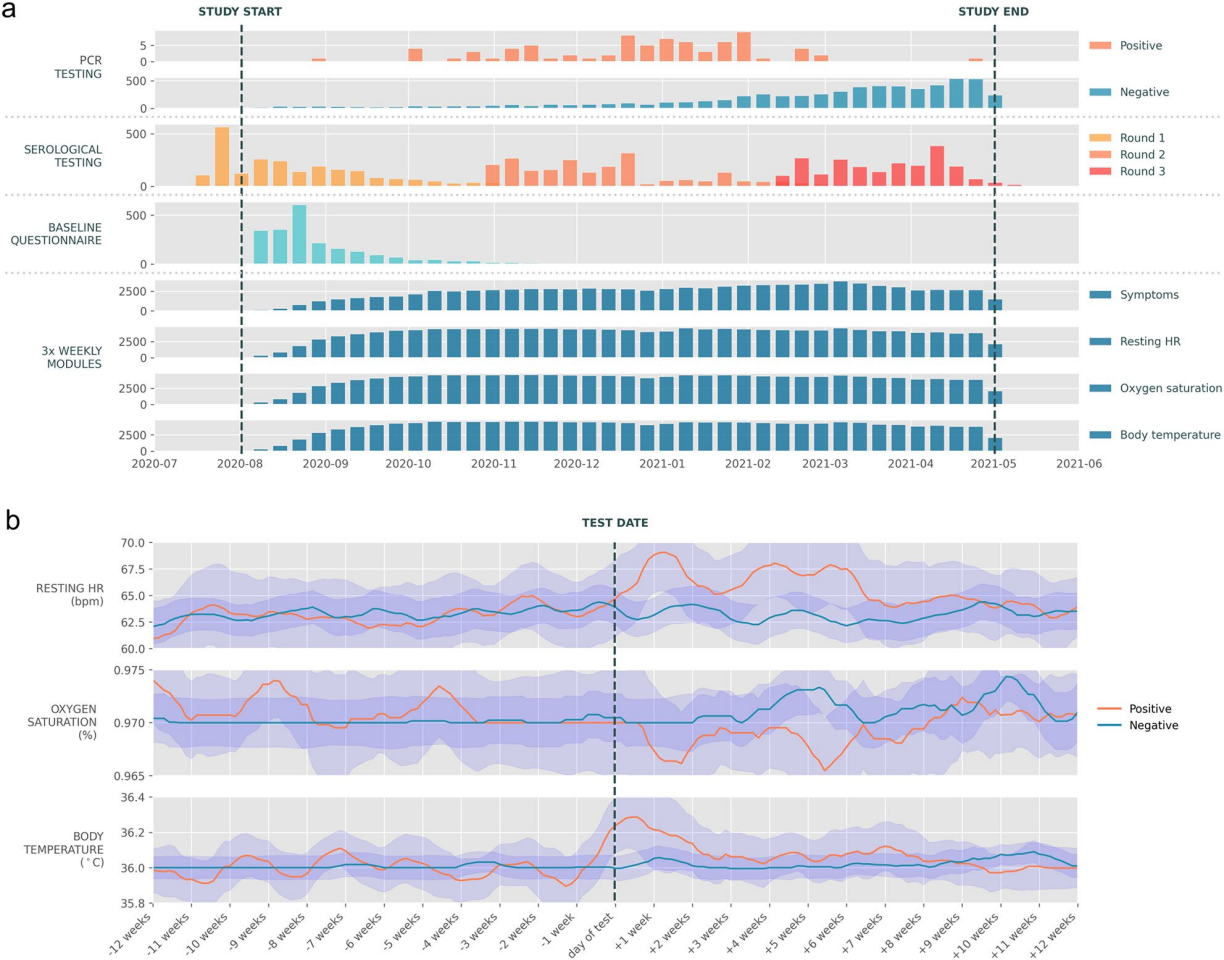
Summary of collected data. (**a**) Timeline of data collection for PCR and antibody/serological testing, baseline questionnaire, symptom questionnaire, and vital signs. Each bar represents the number of records in a particular week of the study. Data for all 2199 participants are shown. (**b**) Seven-day moving average of median vital sign records for positive (n = 33) and negative (n = 304) example participants included in the study around the time of the positive/negative SARS-CoV-2 PCR test (± 12 weeks), 95% confidence intervals included.

In total, there were 2199 participants in the Fenland COVID-19 Huma App study who completed the baseline questionnaire. The majority of positive SARS-CoV-2 PCR tests were recorded in the winter period while the number of negative tests steadily rose over the course of the study as testing became more widely available (Fig. 1a). In this participant pool, there were a total of 77 positive and 6,339 negative SARS-CoV-2 PCR tests during the study period. From the negative tests pool, 304 were then sampled to achieve a balanced dataset, as described in Methods. After applying inclusion and exclusion criteria, 33 positive and 113 negative participants were included in the analysis here (Supplementary Fig. 1a and b). Censoring events are described in Supplementary Fig. 1c.

There were no statistically significant differences between the average vital sign values and demographics of positive (pre-infection) and negative groups (Supplementary Table 4). Visual comparison of data collected on positive and negative groups around the time of SARS-CoV-2 PCR test can be found in Fig. 1b.

### Raw versus transformed input features

As primary input features, we used resting heart rate, oxygen saturation, and body temperature. While daily step data were available, we decided not to use this as the self-isolation policies during the periods of the study would not distinguish between a drop in physical activity due to physiology and the effect of having to self-isolate.

We first supplied the features to the model raw, with one non-transformed record per vital sign per day. This resulted in a cross-validated ROC AUC of 0.538 ± 0.124, precision of 0.305 ± 0.172 and recall of 0.304 ± 0.168. Looking closer at the features which were selected using recursive feature elimination in the five folds, there is no obvious pattern to which features (heart rate vs. oxygen saturation vs. temperature) nor time point before testing that the model found useful for the predictions (Supplementary Fig. 2a). Overall, the poor performance of models trained on the raw features suggests that they are not robust enough for meaningful classification. It is likely that these features are strongly influenced by noise and physiological factors.

As we used simple binary classifiers, we considered the importance of feature transformations on the longitudinal vital sign data. We implemented four different feature transformations: z-score, Isolation Forest, Vector AutoRegression, and maximum over transform days. Using these transformed features instead of the raw inputs resulted in a significant increase of predictive performance, with ROC AUC rising from 0.538 ± 0.124 to 0.695 ± 0.045 (Supplementary Fig. 2b, p = 0.045, two-tailed t-test). The precision of the model with transformed features was 0.465 ± 0.104 and recall 0.601 ± 0.138.

### Impact of varying baselining duration

Larger baseline windows increase the amount of data available to the model when calculating a baseline. However, the increased number of entries is also associated with a much higher variance. Table 1 below summarises the number of oxygen saturation data entries submitted for different baseline window sizes. It should be noted that the vast majority of data submissions involved the participant entering readings for all three vital signs. Other types of data submissions followed the same pattern of increasing variance with longer baseline periods.

**Table 1:** Mean and standard deviation of number of data entries for oxygen saturation.

Baseline window size	Number of data entries	
	Mean	Standard deviation
4 weeks	11.42	4.71
8 weeks	20.87	9.09
12 weeks	25.76	15.38

**Figure 2:**
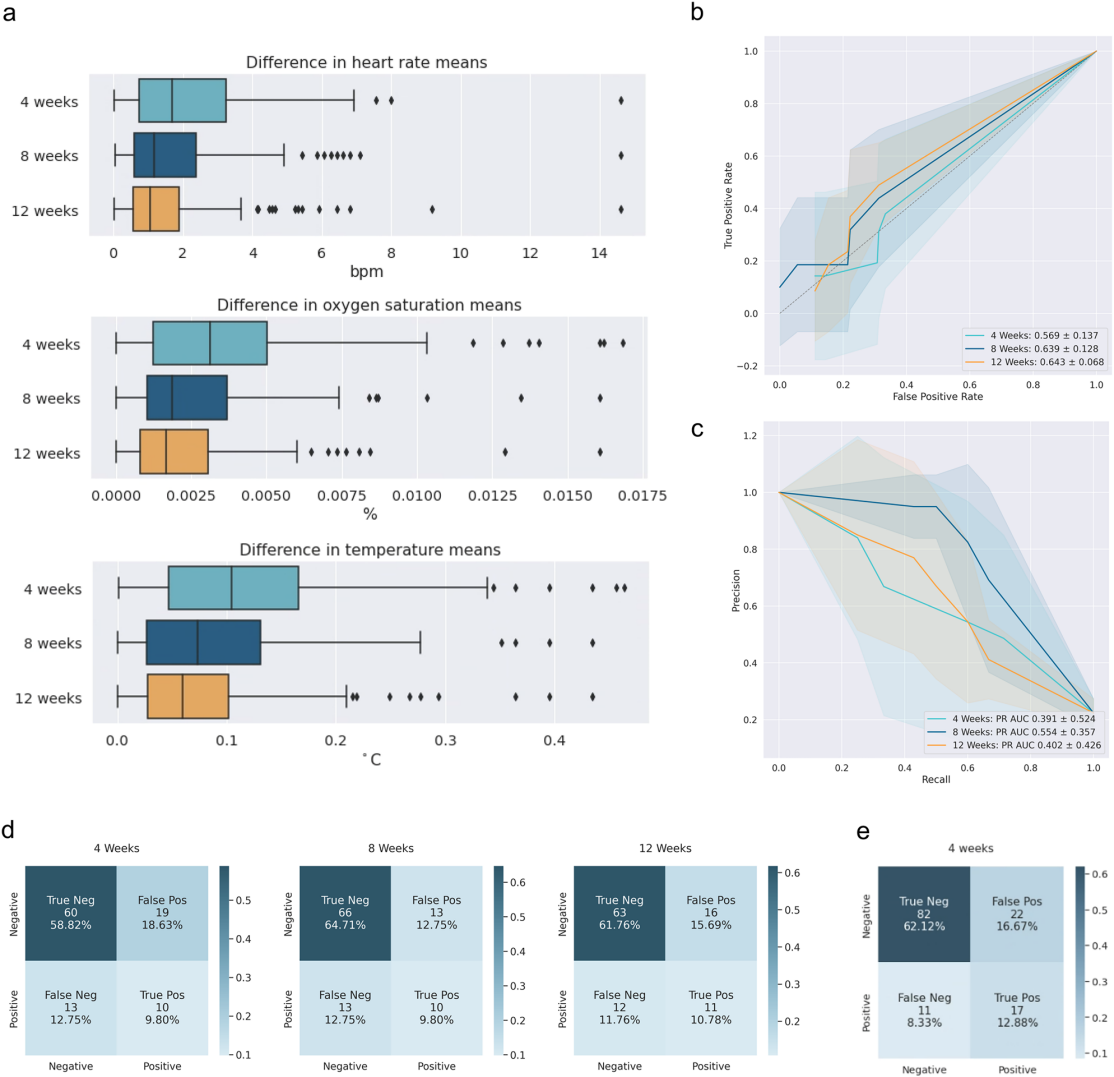
Optimisation of feature pre-processing. (**a**) Absolute differences between baselines calculated using 4, 8, and 12 weeks of measurements and a long-term baseline calculated from all available values for heart rate, oxygen saturation, and body temperature. Each data point corresponds to a participant included in the study. (**b**) ROC curves for models using 4 vs. 8 vs. 12 weeks of input data. (**c**) Precision-Recall curves for models using 4 vs. 8 vs. 12 weeks of input data. (**d**) Confusion matrices with summed values from five folds, along with percentages of total cases included in the test sets. (**e**) Confusion matrix for a model trained on all available data using a 4 week baseline window. In (**b**) and (**c**), the solid line shows the mean of the ROC curves from five folds, and the filled area covers ± one standard deviation of the ROC curves from five folds.

In terms of accuracy of the baselines built on 4, 8 or 12 weeks of data, 4 weeks provided sufficient data to create a reliable baseline. Figure 2a illustrates this, showing that there is an average error of 0.1 °C in temperature between the baseline data collected over 4 weeks, while the average increase in temperature in SARS-CoV-2 positive participants was over 0.2 °C. Still, however, as baseline durations increase, the error in temperature measurements does decrease.

The ROC (Fig. 2b) and Precision-Recall (Fig. 2c) curves are comparable for the 4-, 8- and 12-week baselines, as well as the performance evaluated in the cross-validation pipeline (one-way ANOVA, F(2) = 0.80, p = 0.472)). This suggests that 4 weeks of data is sufficient to provide a reliable baseline for anomaly detection transformations. Confusion matrices obtained by training models with different baseline windows on the same cohort are presented in Fig. 2d. They reinforce the finding that the performance across the three different baseline windows is highly similar. As the window size increases, more participants who tested positive for COVID-19 early in the study have to be excluded. Figure 2e shows the confusion matrix of a 4-week baseline model trained using all available data, showcasing improvement of performance with increased data availability.

### Addition of demographic features or symptoms

We further hypothesised that if the physiological response to SARS-CoV-2 infection differs depending on the age and sex of the infected individual, the addition of these demographic features may improve the discriminative ability of the model. We also had access to self-reported symptom records during the week of the PCR test for 76% of the participants included in the analysis. We evaluated whether the addition of symptom information could further boost model performance. The distribution of the included SARS-CoV-2 symptoms in the analysed population is shown in Supplementary Fig. 3a. There was no statistical difference in the ROC AUC score for the original model (0.695 ± 0.045) and the model which included demographic features (0.660 ± 0.105, p = 0.56, two-tailed t-test, Supplementary Fig. 3b) or symptom features (0.705 ± 0.141, p = 0.94, two-tailed t-test, Supplementary Fig. 3c).

### Prediction of SARS-CoV-2 PCR test positivity using smartphone-collected vital signs

Based on the previous analyses, the pipeline was set up with the use of transformed features for resting heart rate, oxygen saturation, and body temperature, while supplying 4 weeks of data. As described in the Methods section, the unbiased performance was calculated using the pipeline with five fold cross-validation while the final model was generated using the whole dataset (without any hold-out test set).

The final model selected by the optimal parameter search pipeline was a support vector machine classifier with linear kernel and regularisation parameter C of 1000. During the cross-validation, the models selected in the five folds varied (Table 2). Furthermore, the pipeline selected 7 days to be the optimal number of transformed feature days (i.e., of the 4 weeks of total data provided, 21 days were used to generate the baseline, 7 days transformed as input features for the model). This was also the most commonly selected option in the five fold cross-validation.

**Table 2:** Summary of the final model parameters, features and performance.

	Cross-validation (unbiased performance from five folds)	Final model (trained on whole dataset)
**Model parameters**		
Model	3 × LR, 1 × RF, 1 × SVC	SVC
Transform days	1 × 3 days, 3 × 7 days, 1 × 10 days	7 days
**Features**		
Z-score	5 × exclude	Exclude
Isolation forest	1 × include, 4 × exclude	Exclude
VAR	3 × include, 2 × exclude	Exclude
Maximum over transform days	4 × include, 1 × exclude	Include
**Performance**		
ROC AUC in training	0.762 ± 0.068	0.782
ROC AUC on test	0.695 ± 0.045	–
Recall on test	0.601 ± 0.138	–
Precision on test	0.465 ± 0.104	–
Accuracy on test	0.751 ± 0.052	–

Five fold cross-validation results and final model results are shown alongside in columns.

*LR* logistic regression, *RF* random forest, *SVC* support vector classifier, *VAR* vector autoregression.

Regarding transformed features selected as inputs, the final model included only the eight features from the “maximum over transform days”. In the cross-validation, this was also the most commonly used feature set, with four out of five folds selecting it, while VAR was selected three times and the Isolation Forest feature twice. Z-score, which was the simplest transformation of all, was never selected (Table 2). Final model parameters and performance are presented in Table 2.

### Detection of positive SARS-CoV-2 tests in advance of positive test date

**Figure 3:**
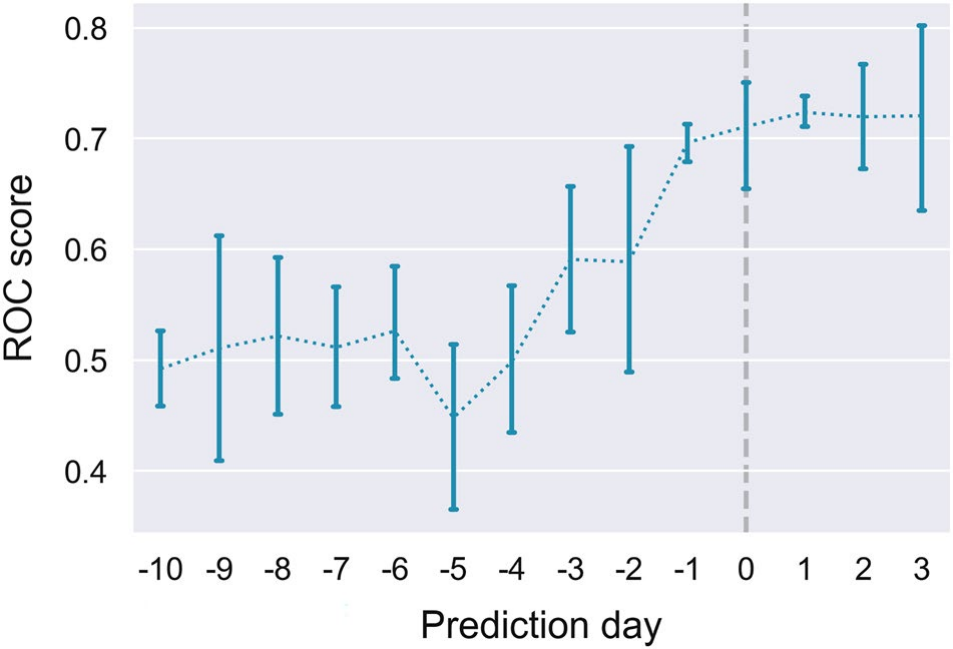
Model performance in predicting PCR test positivity at different days in advance of the positive test date. Day 0 corresponds to the day of the PCR test. ROC AUC score of 0.5 corresponds to a random guess prediction. Mean ± SD shown.

To explore how model performance would change over the course of infection, we trained new models using the previously determined optimal model hyperparameters (Table 2) and data censored at different number of days prior to the positive test. Performance accuracy across the five outer folds is summarised in Fig. 3. The models performed significantly better than random chance when censored from up to 3 days before a positive test. We also found that performance did not improve when using data from the days following a confirmed infection.

### Detection of other events accompanied by similar vital sign changes

**Figure 4:**
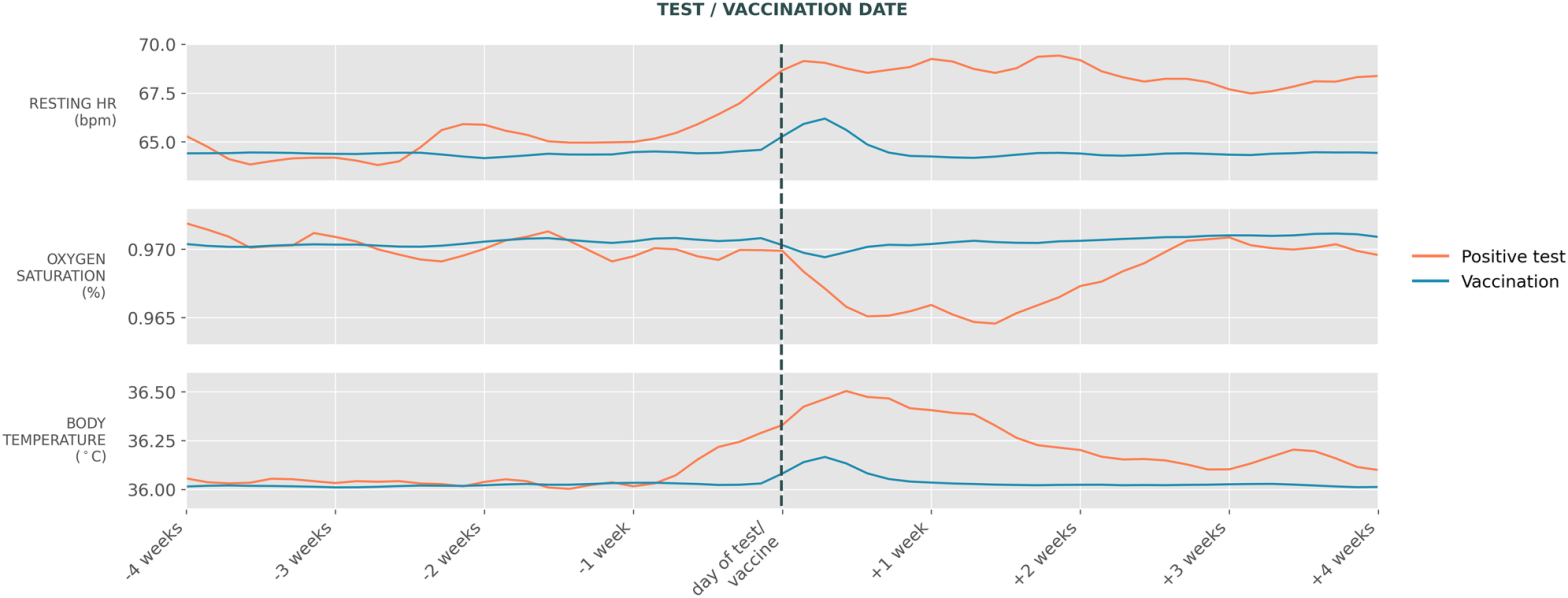
Vital sign changes around the positive SARS-CoV-2 PCR test and COVID-19 vaccination. Three-day moving average of vital sign records shown for ± 4 weeks of data around the test/vaccination date.

We further investigated if our models could predict vaccination events since the vital sign changes after vaccination follow a similar pattern to a real infection, albeit smaller in magnitude (Fig. 4). We applied the same inclusion criteria as in the main study and used the date of first COVID-19 vaccination instead of the positive test date; participants used as negative examples must not have been vaccinated during the window. Positive and negative examples were matched on age and sex. The ROC AUC score of the final model was 0.754, with 0.818 accuracy. As shown in Table 3 summarising precision and recall, the model performs particularly well when predicting negative cases.

**Table 3:** Model performance for prediction of vaccination events.

	Precision	Recall
Unvaccinated	0.92	0.85
Vaccinated	0.47	0.66

Precision and recall are shown for the positive and negative class.

## Discussion

In this study, we present a proof-of-concept classification model for predicting SARS-CoV-2 infection from vital sign data entered on a smartphone in the days preceding a positive SARS-CoV-2 PCR test. While other studies have achieved similar aims, they have focused on use of data from continuously worn specialised wearable devices^17,24,38^. In this pilot study, we present models that require only a smartphone, thermometer, and pulse oximeter. Although our model performance is modestly lower than those based on wearable data, we observed high participant engagement with entering these simple three-times-a-week measurements on the smartphone, which demonstrates the wider potential application of this approach to general population studies^39^. As this method only requires readily available and inexpensive standard devices, it would allow rapid deployment in the general population during infectious disease outbreaks.

Continuous, passive monitoring, such as through wearable devices, although an attractive solution, also has limitations. In some circumstances, measurement bias can be introduced, such as when individuals do not wear their device when feeling unwell^17,40^. There are also issues with compatibility between iOS and Android devices. In our study, implementation of the smartphone app on both iOS and Android devices increased the applicability of the study to the general population. Step counts, as a measure of physical activity, were not used as an input in our model, unlike previous research using activity collected by smartphone or wearable devices^17,18,24,38^. We made this decision because step data could have been influenced by periods of governmental social restriction or shelter-in-place instructions that were widespread as part of the response to SARS-CoV-2^41,42^. It would be difficult to distinguish between a decrease in steps as a result of individuals remaining at home due to these restriction periods over the potential impact of a physiological response to an infection or illness.

The primary outcome was a classification model with a mean five fold cross-validated ROC AUC of 0.695 indicating fair performance. Models based on raw longitudinal vital sign data performed worse than models built with transformed longitudinal vital sign data. A probable reason is that in this remote population-based study, the actual date of infection is unknown and PCR tests may not be undertaken on the actual date of infection. It also demonstrates the utility of including feature transformations as a preprocessing step before classification. By including metrics that summarise the change in individual’s vital signs around the test date the classification task has been made significantly easier. Perhaps if the training dataset were large enough, the model would be able to identify these signals unaided, and these transformations might not be necessary. Nonetheless, our results suggest that these transformations are an efficient strategy when large amounts of training data are not available.

We also demonstrate that adding demographic or symptom data did not improve model performance. This suggests that intermittent vital sign information alone is sufficient for early detection of potential infection. While clinical symptom occurrence certainly carries meaningful information, these may manifest later than vital sign changes and are experienced and recorded subjectively by each individual. This could explain why inclusion of symptoms did not improve the discrimination metrics of the model.

Interestingly, there was no difference in the performance of classifiers built on 4, 8, or 12 weeks of vital sign data. This suggests that a shorter baseline period may be suitable in real-world settings. This dataset was relatively sparse in terms of data points per participant. While increasing measurement density may allow for shorter baseline periods, it should be considered carefully. More frequent monitoring may increase user burden and lead to poorer user engagement.

Our model was able to distinguish between vaccinated and non-vaccinated participants. This suggests that the method of using changes in vital signs to detect infection events is applicable not only to this specific condition and cohort, but could potentially be used to detect other events with similar physiological signatures, such as other influenza-like illnesses.

## Limitations and future scope

The main limitation of this study is the small number of recorded positive cases which limited our decisions around modelling. Due to this small sample size, all positive cases were included in the nested cross-validation without leaving out an unseen test set for validation. Moreover, we had to use heuristics when setting certain thresholds and parameters as we were wary of trying to optimise too many hyperparameters using the automated pipeline on such a small dataset. This study was designed as a proof-of-concept, and it would require a larger dataset to address some of these limitations before being applied in the population at scale.

It should be noted that, at this stage of the pandemic, displaying one of the following symptoms was a requirement for access to a PCR test in the UK: persistent cough, fever, and/or change in smell or taste. As a result, predictions made in the days running up to a positive test cannot be considered “pre-symptomatic”. However, it is still useful to alert individuals that a test or self-isolation may be required. This is the case at the beginning of symptom presentation, when symptoms are mild and not necessarily indicative of COVID-19 infection. Consequently, such early alert systems may lead to earlier testing and detection within the population, reducing the spread of the disease. In the future, it could potentially supplement testing as a method of population surveillance.

Future research to build on this pilot study is required to better align the participant data in relation to the actual infection date. In a general population setting, this date is usually unknown but could potentially be estimated. For example, a probabilistic estimation of the infection date based on known incubation times of the virus and symptom onset could be used, similar to the approach utilised in Hellewell et al.^43^^.^. We did not attempt this approach due to the low availability of accurate symptom data in this pilot study.

## Conclusion

In conclusion, we present a pilot study for predicting symptomatic SARS-CoV-2 confirmed with a positive SARS-CoV-2 PCR test using only vital sign data. The model had fair performance and provides evidence for the utility of user-collected, smartphone-entered data in detecting physiological changes associated with the early stages of respiratory infections. It could be developed further for use in remote as well as resource-poor settings for early detection of respiratory infections.

## Supplementary Information

Supplementary Information.

## Data Availability

Data is available on reasonable request to the authors. Details of the investigators involved in this study can be found here: https://www.mrc-epid.cam.ac.uk/research/studies/fenland-covid19/researcher-info/. For more information on data sharing please visit: https://www.mrc-epid.cam.ac.uk/research/data-sharing/.

## Code availability

Code is available on reasonable request to the authors.

## References

[1] Thornton J (2020). The “virtual wards” supporting patients with covid-19 in the community. BMJ Br. Med. J..

[2] Shah S (2020). Novel use of home pulse oximetry monitoring in COVID-19 patients discharged from the emergency department identifies need for hospitalization. Acad. Emerg. Med..

[3] Lim A (2022). An outpatient management strategy using a coronataxi digital early warning system reduces coronavirus disease 2019 mortality. Open Forum Infect. Dis..

[4] Menni C (2022). Symptom prevalence, duration, and risk of hospital admission in individuals infected with SARS-CoV-2 during periods of omicron and delta variant dominance: A prospective observational study from the ZOE COVID Study. Lancet.

[5] Josan K (2021). Validation of a pandemic-proof, decentralized cardiovascular trial: Scalable design produces rapid recruitment, high engagement and protocol adherence in DeTAP (Decentralized Trial in Afib Patients). Eur. Heart J..

[6] Indraratna P (2021). Trials and tribulations: mHealth clinical trials in the COVID-19 pandemic. Yearb. Med. Inform..

[7] UK Health Security Agency. Coronavirus (COVID-19) in the UK. https://coronavirus.data.gov.uk/details/cases?areaType=overview&areaName=United%20Kingdom, (Accessed 31 May 2023).

[8] Our World in Data. SARS-CoV-2 variants in analyzed sequences, United Kingdom. https://ourworldindata.org/grapher/covid-variants-area?time=2020-08-03..2021-04-26&country=~GBR (Accessed 31 May 2023).

[9] Lauer SA (2020). The incubation period of coronavirus disease 2019 (COVID-19) from publicly reported confirmed cases: Estimation and application. Ann. Intern. Med..

[10] McAloon C (2020). Incubation period of COVID-19: A rapid systematic review and meta-analysis of observational research. BMJ Open.

[11] He X (2020). Temporal dynamics in viral shedding and transmissibility of COVID-19. Nat. Med..

[12] Hart WS, Maini PK, Thompson RN (2021). High infectiousness immediately before COVID-19 symptom onset highlights the importance of continued contact tracing. Elife.

[13] Byrne AW (2020). Inferred duration of infectious period of SARS-CoV-2: Rapid scoping review and analysis of available evidence for asymptomatic and symptomatic COVID-19 cases. BMJ Open.

[14] Mehta OP, Bhandari P, Raut A, Kacimi SEO, Huy NT (2021). Coronavirus disease (COVID-19): Comprehensive review of clinical presentation. Front. Public Health.

[15] Booth A (2021). Population risk factors for severe disease and mortality in COVID-19: A global systematic review and meta-analysis. PLoS ONE.

[16] Jarvis KF, Kelley JB (2021). Temporal dynamics of viral load and false negative rate influence the levels of testing necessary to combat COVID-19 spread. Sci. Rep..

[17] Mishra T (2020). Pre-symptomatic detection of COVID-19 from smartwatch data. Nat. Biomed. Eng..

[18] Natarajan A, Su H-W, Heneghan C (2020). Assessment of physiological signs associated with COVID-19 measured using wearable devices. NPJ Digit. Med..

[19] Smarr BL (2020). Feasibility of continuous fever monitoring using wearable devices. Sci. Rep..

[20] Radin JM, Wineinger NE, Topol EJ, Steinhubl SR (2020). Harnessing wearable device data to improve state-level real-time surveillance of influenza-like illness in the USA: A population-based study. Lancet Digit. Health.

[21] Li X (2017). Digital health: Tracking physiomes and activity using wearable biosensors reveals useful health-related information. PLoS Biol..

[22] Shapiro A (2021). Characterizing COVID-19 and influenza illnesses in the real world via person-generated health data. Patterns.

[23] Corcoran SE, O’Neill LAJ (2016). HIF1α and metabolic reprogramming in inflammation. J. Clin. Investig..

[24] Hirten RP (2021). Use of physiological data from a wearable device to identify SARS-CoV-2 infection and symptoms and predict COVID-19 diagnosis: Observational study. J. Med. Internet Res..

[25] Dabbah MA (2021). Machine learning approach to dynamic risk modeling of mortality in COVID-19: A UK Biobank study. Sci. Rep..

[26] Mitratza M (2022). The performance of wearable sensors in the detection of SARS-CoV-2 infection: A systematic review. Lancet Digit. Health.

[27] Lindsay T (2019). Descriptive epidemiology of physical activity energy expenditure in UK adults (The Fenland study). Int. J. Behav. Nutr. Phys. Act..

[28] Koulman, A. *et al.* The development, validation and application of remote blood sample collection in telehealth programmes. *J. Telemed. Telecare* **0**, (2022). 10.1177/1357633X221093434 PMC11027437 35538704

[29] World Medical Organisation. WMA Declaration of Helsinki - Ethical Principles for Medical Research Involving Human Subjects. https://www.wma.net/policies-post/wma-declaration-of-helsinki-ethical-principles-for-medical-research-involving-human-subjects/ (Accessed 31 May 2023).

[30] Mol D (2020). Performance of an automated photoplethysmography-based artificial intelligence algorithm to detect atrial fibrillation. Cardiovasc. Digit. Health J..

[31] Pedregosa F (2011). Scikit-learn: Machine learning in Python. J. Mach. Learn. Res..

[32] Seabold, S. & Perktold, J. statsmodels: Econometric and statistical modeling with python. In *9th Python in Science Conference* (2010).

[33] Akiba, T., Sano, S., Yanase, T., Ohta, T. & Koyama, M. *Optuna: A Next-generation Hyperparameter Optimization Framework*. (ACM, 2019). 10.1145/3292500.3330701.

[34] Vabalas A, Gowen E, Poliakoff E, Casson AJ (2019). Machine learning algorithm validation with a limited sample size. PLoS ONE.

[35] Pollard TJ, Johnson AEW, Raffa JD, Mark RG (2018). tableone: An open source Python package for producing summary statistics for research papers. JAMIA Open.

[36] Hunter JD (2007). Matplotlib: A 2D graphics environment. Comput. Sci. Eng..

[37] Waskom ML (2021). seaborn: Statistical data visualization. J. Open Source Softw..

[38] Alavi A (2022). Real-time alerting system for COVID-19 and other stress events using wearable data. Nat. Med..

[39] Rennie KL (2023). Engagement with mHealth COVID-19 digital biomarker measurements in a longitudinal cohort study: Mixed methods evaluation. J. Med. Internet Res..

[40] Shandhi MMH (2022). A method for intelligent allocation of diagnostic testing by leveraging data from commercial wearable devices: A case study on COVID-19. Npj Digit. Med..

[41] Eshelby V, Sogut M, Jolly K, Vlaev I, Elliott MT (2022). Stay home and stay active? The impact of stay-at-home restrictions on physical activity routines in the UK during the COVID-19 pandemic. J. Sports Sci..

[42] Panicker RM, Chandrasekaran B (2022). ‘Wearables on vogue’: A scoping review on wearables on physical activity and sedentary behavior during COVID-19 pandemic. Sport Sci. Health.

[43] Hellewell J (2021). Estimating the effectiveness of routine asymptomatic PCR testing at different frequencies for the detection of SARS-CoV-2 infections. BMC Med..

