# Supplementary material for: Feasibility of using intermittent active monitoring of vital signs by smartphone users to predict SARS-CoV-2 PCR positivity
Source: Sci Rep. 2023 Jun 29;13:10581. doi: 10.1038/s41598-023-37301-y (PMC10310739; doi:10.1038/s41598-023-37301-y)
Supplement: Supplementary file 1 — Supplementary Information. [file 41598_2023_37301_MOESM1_ESM.pdf]

Supplementary Materials

Feasibility of using intermittent active monitoring of vital signs by smartphone users to predict SARS-CoV-2 PCR positivity

Authors: Nikola Dolezalova, Effrossyni Gkrania-Klotsas, Davide Morelli, Alex Moore, Adam C Cunningham, Adam Boot[1], David Plans, Angus B Reed, Mert Aral, Kirsten L Rennie, and Nicholas J Wareham.

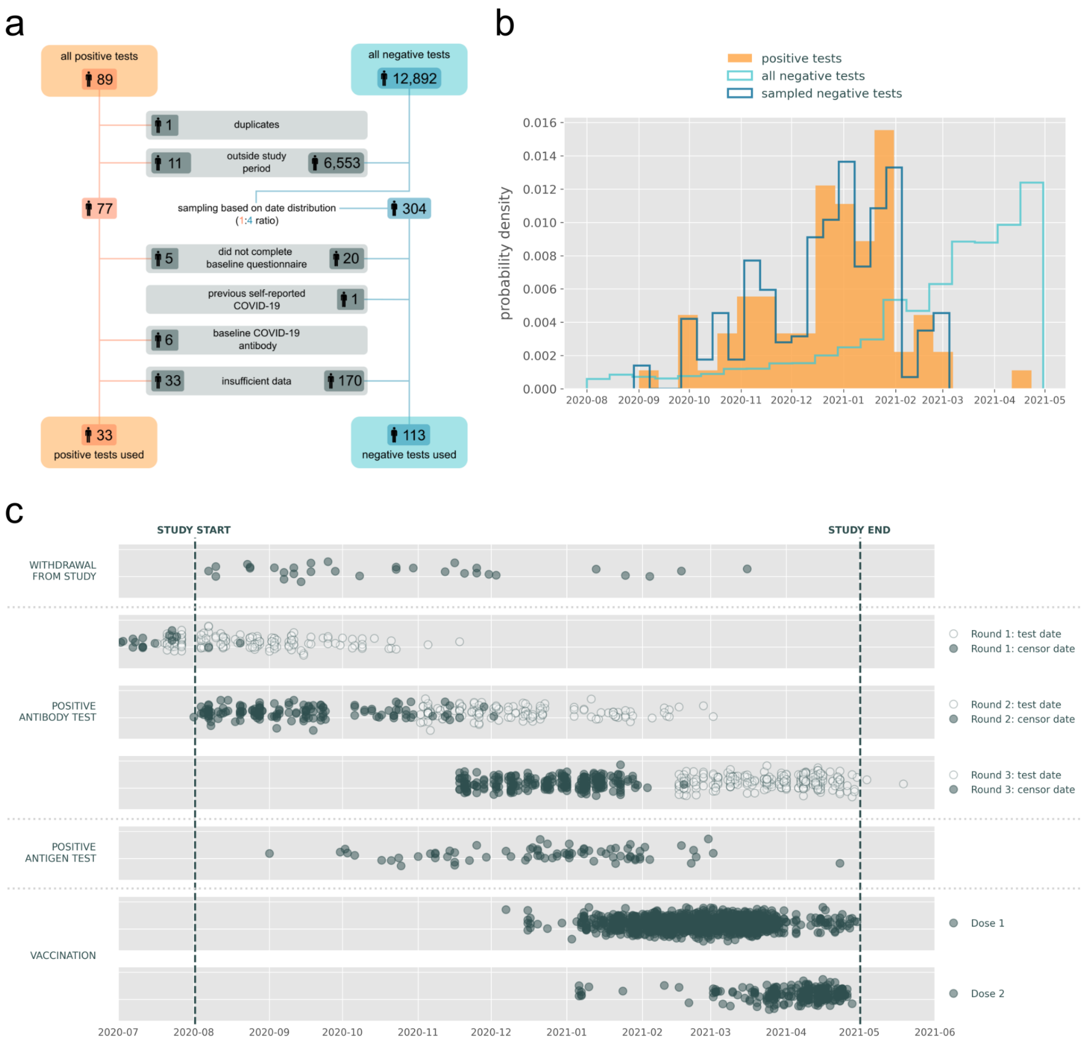

***Supplementary Figure 1**: **Details of participants affected by inclusion and exclusion criteria, and censoring.** a) Flow of participants through the study. **b)** Distribution of PCR tests over the time of the study. There were 77 positive tests (orange), majority of which were in the winter period. The total number of negative tests was 6339 (light blue) and their number grew towards the end of the study as testing became more available to the general population. For the classification task we sampled 304 negative tests (dark blue, approximately 4:1 ratio with the positive tests) to correspond with the time distribution of the positive tests. **c)** Summary of censoring events for all participants in the study. Each point is a single censoring event date, for antibody tests, empty circles mark the date of the positive test and full circles show the actual censoring date which comes 90 days before the test result. Censoring was applied from the censoring date shown until the end of the study with the exception of vaccination where data was censored for 5 days following the vaccination date.*

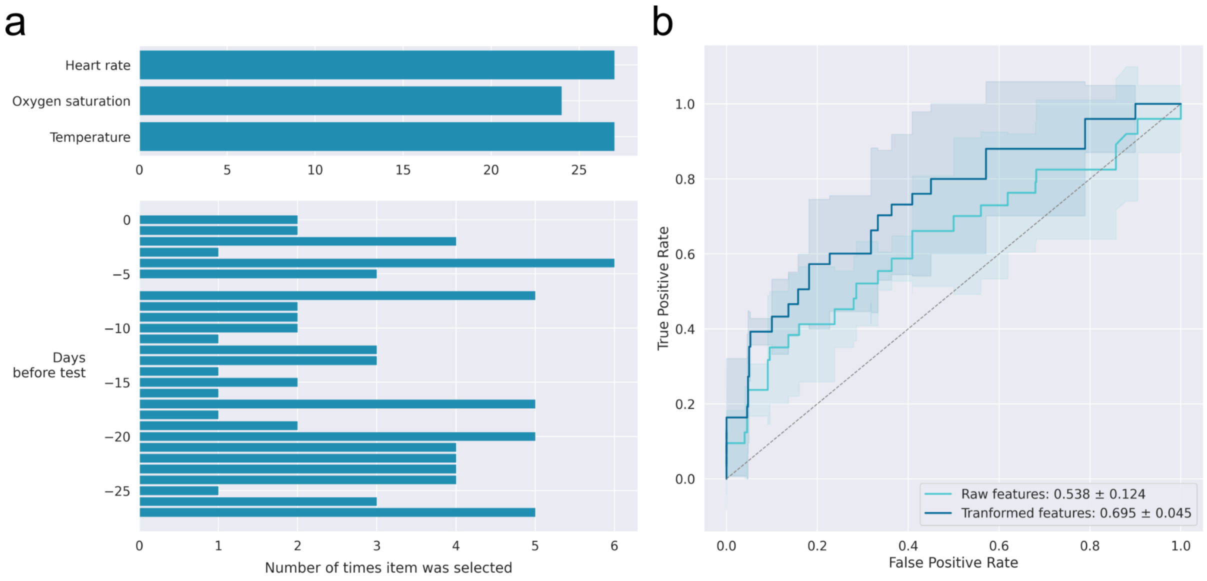

Supplementary Figure 2: Optimisation of feature pre-processing. a) *Analysis of most selected raw features during feature selection in the five-fold cross-validation. During training the model sub selected the most discriminative features at various time points (relative to test date). Features are grouped by the vital sign (top) and number of days before SARS-CoV-2 antigen test (bottom).* ***b)*** *Comparison of performance with and without transformed features. ROC curves for models using raw vs. transformed features. The solid line shows the mean of the ROC curves from 5 folds, and the filled area covers ± one standard deviation of the ROC curves from 5 folds.*

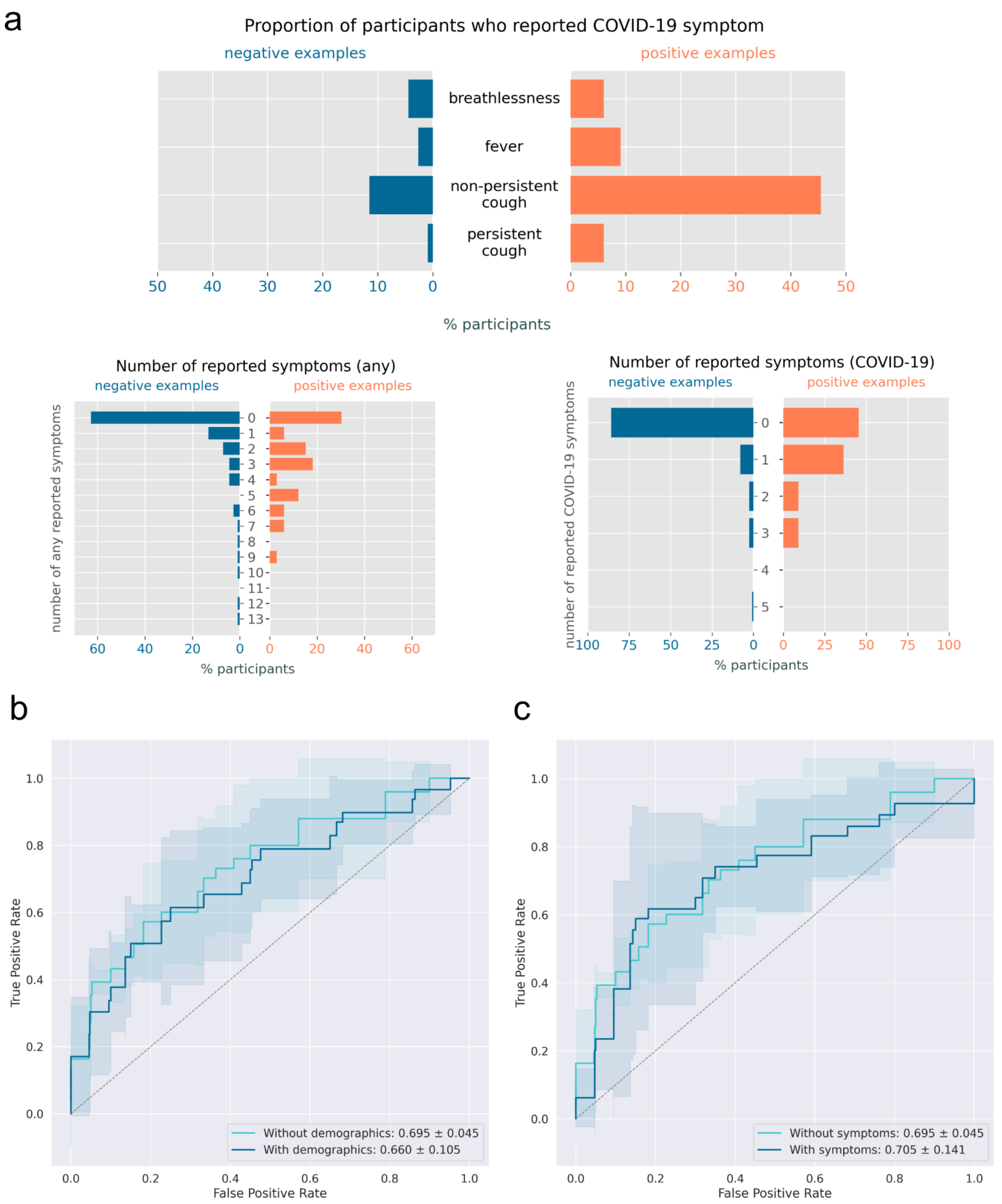

Supplementary Figure 3: Effect of further feature addition on model performance. a) *Summary of collected symptom data per participant in the study. Proportion of participants reporting particular symptoms (top) and number of reported symptoms in the 7 days prior to the SARS-CoV-2 antigen test (bottom). Participants who did not complete the Symptoms module and participants who reported not having symptoms are grouped in this analysis. List of symptoms can be found in Supplementary Table 1.* ***b)*** *ROC curves for models without and with demographics features (age and sex).* ***c)*** *ROC curves for models without and with symptom features (binary features for breathlessness, fever, persistent or non-persistent cough in the 7 days before test, number of SARS-CoV-2 symptoms (named earlier) in 7 days before test, number of times any symptoms reported in the 7 days before test). In b) and c), the solid line shows the mean of the ROC curves from 5 folds, and the filled area covers ± one standard deviation of the ROC curves from 5 folds.*

Supplementary Table 1: Features used in the model and any processing/transformation steps. *The actual number of variables in the model was based on window length for raw features (how many days of data is provided to the model) and the number of days to transform (how many days out of the window length to use for transformation rather than calculation of the baseline) for transformed features. In addition to this, raw features were subjected to recursive feature selection, transformed features were selected during the Optuna hyperparameter optimization in sets (see Supplementary Table 2)*

Feature	Description	Number of features
**Raw features**		**3 x window length***
Resting heart rate	Non-physiological values & outliers removed, one value per day (mean) taken, up-sampled on a daily basis (linear interpolation to fill in missing values)	One for each day
Oxygen saturation		One for each day
Temperature		One for each day
**Transformed features**		**(8 x transform days**) + 8**
**Z-score**		**3 x transform days****
Z-score: resting heart rate	Mean and standard deviation over the baseline period were calculated for each participant and Z-score was calculated for each day of the transform period	One for each day
Z-score: oxygen saturation		One for each day
Z-score: temperature		One for each day
**Isolation forest score**		**1 x transform days****
Isolation forest: multivariate	Isolation Forest model was trained for each participant on the baseline period data and predictions/scores issued for every day of the transform period	One for each day
**VAR reconstruction error**		**4 x transform days****
VAR: resting heart rate	VAR model was trained for each participant on the baseline period, forecast was issued for each day of the transform period and reconstruction error calculated	One for each day
VAR: oxygen saturation		One for each day
VAR: temperature		One for each day
VAR: multivariate	Sum of the VAR reconstruction errors for all three vital signs (normalised by dividing by mean of each variable to have comparable impact on the multivariate score)	One for each day
**Maximum over transform days****		**8**
Maximum: Z-score	For each transformed feature, the maximum value (i.e. the most abnormal measurement) over the transform period was taken	3 (one for every z-score variable)
Maximum: Isolation forest		1 (multivariate only)
Maximum: VAR		4 (one for every VAR variable)
**Additional features**		
Age	Age in years at the start of the study (01/08/2020)	1
Sex	Binary feature (male=0, female=1)	1
Symptoms	Extracted two numerical features: Number of symptoms reported in the 7 days prior to	6

	SARS-CoV-2 antigen test (any symptoms*** or selected SARS-CoV-2 symptoms****)<br><br>Extracted four binary features for whether participant reported a selected SARS-CoV-2 symptom**** in the 7 days prior to SARS-CoV-2 antigen test.

** the number of days of data supplied to the model (a pre-set parameter of the model), 4, 8 or 12 weeks of data were tested in this study*

*** the number of days from the window length to transform (an optimizable parameter of the model), can obtain values 3, 7, 10 or 14*

**** full list of symptoms: feeling sick, diarrhoea, nasal discomfort, breathlessness, fever, headache, hoarseness, joint ache, loss of appetite, loss of taste or smell, muscle ache, non-persistent cough, persistent cough, sneezing, sore throat, tiredness, wheeze, other*

***** full list of selected SARS-CoV-2 symptoms: breathlessness, fever, non-persistent cough, persistent cough*

Supplementary Table 2: Search space for Optuna hyperpameter optimisation. *Two groups of hyperparameters are included: those related to feature space and those related to classification models. The number of potential configurations was then used to inform the number of Optuna trials in each experiment to cover a sufficient proportion of configurations.*

Parameter	Search space	Potential configurations
**Feature space: Transformed features**		**64**
Days to transform	3, 7, 10, 14	4
Include Z-score features	Exclude, include	2
Include Isolation Forest features	Exclude, include	2
Include VAR features	Exclude, include	2
Include maximum over transformed days	Exclude, include	2
**Model: Logistic regression**		**6**
C	0.001, 0.01, 0.1, 1, 10, 100	6
**Model: Random Forest**		**30**
Number of estimators	16, 32, 64, 128, 256, 512	6
Maximum tree depth	1, 2, 4, 8, 16	5
**Model: Support Vector Classifier**		**28**
C	0.001, 0.01, 0.1, 1, 10, 100, 1000	7
Kernel	Linear, RBF	2
Gamma	Scale, Auto	2
**TOTAL (if raw features)**		**64**
**TOTAL (if transformed features)**		**4,096**

Supplementary Table 3: Python libraries and their versions used in the study.

Library	Version	Citation
scikit-learn	0.24.2	Pedregosa, F., G. Varoquaux, A. Gramfort, V. Michel, B. Thirion, O. Grisel, M. Blondel, et al. 'Scikit-Learn: Machine Learning in Python'. *Journal of Machine Learning Research* 12 (2011): 2825–30.
numpy	1.20.0	Van Der Walt, Stefan, S Chris Colbert, and Gael Varoquaux. 'The NumPy Array: A Structure for Efficient Numerical Computation'. *Computing in Science & Engineering* 13, no. 2 (2011): 22–30. https://doi.org/10.1109/MCSE.2011.37.
pandas	1.1.0	The pandas development team. *Pandas-Dev/Pandas: Pandas* (1.1.0). Zenodo, 2020. https://doi.org/10.5281/zenodo.3509134.
matplotlib	3.2.1	Hunter, J. D. 'Matplotlib: A 2D Graphics Environment'. *Computing in Science & Engineering* 9, no. 3 (2007): 90–95. https://doi.org/10.1109/MCSE.2007.55.
seaborn	0.11.2	Waskom, Michael L. 'Seaborn: Statistical Data Visualization'. *Journal of Open Source Software* 6, no. 60 (2021): 3021. https://doi.org/10.21105/joss.03021.
optuna	2.10.0	Akiba, Takuya, Shotaro Sano, Toshihiko Yanase, Takeru Ohta, and Masanori Koyama. 'Optuna: A Next-Generation Hyperparameter Optimization Framework'. ACM, 2019. https://doi.org/10.1145/3292500.3330701.
multiprocess	0.70.12.2	McKerns, Michael M., Leif Strand, Tim Sullivan, Alta Fang, and Michael A. G. Aivazis. 'Building a Framework for Predictive Science'. arXiv, 6 February 2012. https://doi.org/10.48550/arXiv.1202.1056.<br>Michael McKerns and Michael Aivazis,"pathos: a framework for heterogeneous computing", 2010- ; http://uqfoundation.github.io/project/pathos.
tableone	0.7.10	Pollard, Tom J, Alistair E W Johnson, Jesse D Raffa, and Roger G Mark. 'Tableone: An Open Source Python Package for Producing Summary Statistics for Research Papers'. *JAMIA Open* 1, no. 1 (1 July 2018): 26–31. https://doi.org/10.1093/jamiaopen/ooy012.
statsmodels	0.13.2	Seabold, Skipper, and Josef Perktold. 'Statsmodels: Econometric and Statistical Modeling with Python'. In *9th Python in Science Conference*, 2010.

Supplementary Table 4: Demographic comparison of positive and negative population. *Participant values for longitudinal variables (resting heart rate, oxygen saturation and temperature) were derived as a median of all values recorded over the course of the study. Values of socioeconomic status class were missing for 6 participants. The statistical comparisons of positive and negative populations were obtained either by [a] two-sample t-test or [b] chi square test.*

	Overall	Positive cases	Negative cases	p-value
**n**	**146**	**33**	**113**	
**Resting heart rate, mean (SD)**	64.09 (8.75)	63.91 (8.60)	64.15 (8.83)	1[a]
**Oxygen saturation, mean (SD)**	0.97 (0.01)	0.97 (0.01)	0.97 (0.01)	1[a]
**Temperature, mean (SD)**	36.02 (0.27)	36.04 (0.28)	36.02 (0.27)	1[a]
**Age, mean (SD)**	57.28 (6.94)	56.19 (6.61)	57.60 (7.03)	1[a]
**Sex, n (% female)**	91 (62.33)	20 (60.61)	71 (62.83)	1[b]
**Socioeconomic status class**				1[b]
Professional/higher managerial occupations, n (%)	106 (72.60)	20 (60.61)	86 (76.11)	
Other (lower managerial, clerical, routine, service & technical), n (%)	34 (23.28)	11 (33.33)	23 (20.35)	
